# The Role of Macrophages in Oral Squamous Cell Carcinoma

**DOI:** 10.3389/fonc.2021.611115

**Published:** 2021-03-18

**Authors:** Eleni Marina Kalogirou, Konstantinos I. Tosios, Panagiotis F. Christopoulos

**Affiliations:** ^1^Department of Oral Medicine and Pathology, Faculty of Dentistry, National and Kapodistrian University of Athens, Athens, Greece; ^2^Department of Pathology, Oslo University Hospital and University of Oslo, Oslo, Norway

**Keywords:** oral cancer, oral squamous cell carcinoma, tumor-associated macrophages (TAMs), M1-M2 phenotype, classical activation, macrophage polarization

## Abstract

Oral cancer is a common malignancy worldwide, with high disease-related death rates. Oral squamous cell carcinoma (OSCC) accounts for more than 90% of oral tumors, with surgical management remaining the treatment of choice. However, advanced and metastatic OSCC is still incurable. Thus, emphasis has been given lately in understanding the complex role of the oral tumor microenvironment (TME) in OSCC progression, in order to identify novel prognostic biomarkers and therapeutic targets. Tumor associated macrophages (TAMs) constitute a major population of the OSCC TME, with bipolar role in disease progression depending on their activation status (M1 vs. M2). Here, we provide an up to date review of the current literature on the role of macrophages during oral oncogenesis, as well as their prognostic significance in OSCC survival and response to standard treatment regimens. Finally, we discuss novel concepts regarding the potential use of macrophages as targets for OSCC immunotherapeutics and suggest future directions in the field.

## Introduction

The global incidence of oral cancer was 377,713 new cases during 2020 (1), with a tendency for increasing occurrence even in countries where it is less common, such as Finland and Denmark (2, 3). More than 90% of oral malignancies are oral squamous cell carcinomas (OSCCs), mostly that of the mobile part of the tongue (4, 5). Surgical management is the treatment of choice for OSCC, occasionally supplemented by radiotherapy or chemotherapy. Despite the advances in treatment modalities for solid tumors, the 5-year survival rate is 50% (4, 5), establishing OSCC as the 7th and 10th cause of death for males and females, respectively, in Europe (6).

Prognosis of OSCC depends on various factors associated with patients' profile, i.e., ethnicity, gender, age, socioeconomic status, and lifestyle, mainly including smoking and alcohol consumption, as well as tumor's characteristics, i.e., site, size, regional or distant metastases, stage, depth of invasion and degree of differentiation (5). Most research efforts focus on the identification of novel biomarkers in blood, saliva, or tumor tissue samples that could facilitate early diagnosis and group patients into subpopulations with more predictable responses to certain treatment schemes (5, 7). Such biomarkers might be directly related to tumor cells *per se*, or concern cellular and/or other components of the surrounding tumor microenvironment (TME) (8). TME provides the essential requirements for cancer survival, growth and invasion (8) and macrophages are a key population of it (9).

We review the literature on the role of macrophages in OSCC, focusing on their pro-tumor and anti-tumor properties, as well as their prognostic significance for patients' survival and response to standard treatment regimens. We, also, discuss novel concepts regarding the potential use of macrophages as candidates for OSCC immunotherapy and suggest future directions in the field.

## The Tumor Microenvironment of Oral Cancer

The progression of OSCC depends on the interplay among the cancer cells, the surrounding host-derived stromal cells, e.g., fibroblasts, endothelial cells and pericytes, and the extracellular matrix non-cellular components composing the TME (10). Immune cells may have opposing functions in oral oncogenesis, i.e., a class of macrophages, myeloid-derived suppressor cells, regulatory T cells and CD4+ T helper type 2 (Th2) cells may share pro-tumor functions, whereas another class of macrophages, dendritic cells, natural killer cells, CD8+ T cells and CD4+ Th1 cells may have anti-tumor actions (11). Historically solid tumors are immunologically classified into “*cold”* and “*hot,”* depending on the non-inflamed or inflamed TME milieu, respectively (12, 13). Inflamed tumors respond favorably to immune checkpoint blockade (ICB) therapy and are characterized by an abundance of tumor infiltrated lymphocytes (TILs) enriched for interferon-γ (IFN-γ)-expressing CD8+ T cells, expression of checkpoint markers including programmed death-1 ligand 1 (PD-L1) and high mutational burden. In contrast, non-inflamed tumors are poorly infiltrated by immune cells and rarely express PD-L1, while characterized by an immunosuppressive milieu, though they might also have high genomic instability. On the very end of the tumor immunity continuum are immunologically ignorant tumors, characterized by genomic stability, highly proliferative tumor cells and low infiltration of T cells, as well as low expression of antigen-presentation machinery markers including MHCI (14). Immunogenic tumors, characterized by the successful recognition of cancer antigens, vary (15, 16), but the presence of macrophages in the TME is independent of the tumor's immunogenicity status.

The interplay between the innate and the acquired immune system is responsible for the recognition of cancer specific antigens as foreign antigens, a process termed *immunosurveillance* that eliminates cancer cells (17). Besides its natural protective role against tumors, the identification of a tumor promoting role of the immune system, mainly via clonal selection (those with low immunogenicity), has led to the refinement of the initial immunosurveillance hypothesis (18, 19) with that of cancer immunoediting (20, 21). This process consists of three phases, known as the 3Es of cancer immunoediting; elimination, equilibrium and escape. The elimination phase represents the original immunosurveillance concept and raises as an integrated and combined response of both the innate and adaptive immune system against developing tumors. Complete eradication of tumor cells at this stage means that there is not progression to the following two phases and thus homeostasis has achieved. If that's not the case then in the second phase, which is most probably the longest of the three (may last for years in humans) and the direct equivalent of a Darwinian selection process, the tumor cell variants that had survived the first phase enter a dynamic equilibrium with the immune system, which is able to contain but not completely vanish the tumor variants. The result of this sculpturing is the generation of heterogeneous and genetically unstable tumor clones with increased resistance to immune recognition. In the last phase, the selected tumor cell variants from the equilibrium phase can grow and expand to clinically detectable levels. Even though various genetic events and epigenetic modifications, as well as different direct and indirect mechanisms may be employed by cancer cells, the end result is the tumor escape from host's immune defenses (22, 23). In the first line of immune response are professional antigen presenting cells, e.g., dendritic cells, B cells and macrophages. Macrophages belong to the mononuclear phagocytic system and are the final differentiation stage of circulating monocytes that have been attracted by chemotactic factors to the tissue site, as a response to various microenvironmental stimuli (24, 25). Tissue resident macrophages originate from yolk sac during embryonic development, whereas monocyte-derived macrophages from bone marrow progenitor cells (26). *Tumor-associated macrophages* (TAMs) constitute the major leucocytic component of the OSCC TME (27).

TAMs may have promoting or inhibitory effects on OSCC cancer cells proliferation, invasion and migration (10). They are directed to the tumor site by chemotactic cues produced by cancer cells or stromal cells, including vascular endothelial growth factor (VEGF), colony stimulating factor-1 (CSF-1), placental growth factor, and chemokines, such as the chemokine (C-C motif) ligand (CCL) 2/monocyte chemotactic protein-1, CCL3/macrophage inflammatory protein-1alpha, CCL4/macrophage inflammatory protein-1beta, and the CCL5/regulated on activation, normal T-cell expressed and secreted (RANTES) (28, 29). TAMs are usually more prevalent in tumor stroma than in OSCC cell nests (30).

Initially, TAMs recruited in the hypoxic TME environment may show an anti-tumor activity, e.g., via the tumor-antigen presentation to T cells and the induction of the T-cell mediated cancer cytotoxicity (31). TAMs have been shown to be effective in inducing the cytotoxic activation of naive (CD45RO) or memory T cell (CD45RA) against *Streptococcus salivarius*, while their depletion caused decreased levels of granzyme B expressed by CD8+ T cells (32). On the other hand, TAMs may be a source of proangiogenic molecules and growth factors promoting cell proliferation, such as VEGF, epidermal growth factor (EGF), fibroblast growth factor, platelet-derived growth factor, transforming growth factor beta (TGF-β) and CCL2, as well as matrix metalloproteinases (MMPs), responsible for the degradation of the basement membrane and extracellular matrix, thus facilitating the progression of OSCC (28, 29, 33). The EGF-mediated pro-tumor role of TAMs in OSCC has been highlighted in a study showing increased cell proliferation and invasion of OSCC cells co-cultured with TAMs-derived conditioned medium, while the proliferation and invasion activity was hampered after cancer cell treatments with an anti-EGF receptor antibody (34). In another study, TAMs-induced progression of OSCC was associated with promotion of epithelial-to-mesenchymal transition of OSCC cells, as a fibroblast-like phenotype was observed in an OSCC and two head and neck cell lines following co-culture with TAMs conditioned medium (35).

A critical factor defining the pro-tumor or anti-tumor properties of TAMs is their activation state.

## Activation States of Macrophages

Besides their antigen-presentation capabilities, macrophages possess various other properties necessary to maintain homeostasis, e.g., phagocytosis of pathogens and cell debris, destruction of antibody-coated cells or microbes, secretion of growth factors, cytokines and chemokines, expression of membranous co-stimulatory or co-inhibitory molecules, and wound healing via matrix destruction, regeneration and angiogenesis (36, 37). The wide and contradictory functions of macrophages are represented by the two-edged model of M1 or M2 macrophages, associated with Th1 or Th2 responses, respectively (25, 38). Metabolic events seem to drive the polarization fate of macrophages (39); the differential metabolism of L-arginine, either via inducible nitric oxide synthase (iNOS) to NO and citruline, or via arginase to ornithine and urea, defines the orientation toward M1 or M2 activation, respectively (40).

The M1 macrophage phenotype arises as the consequence of classical activation stimulated by bacterial components, e.g., lipopolysaccharides, or by the Th1-driven cytokine IFN-γ (10, 41). M1 macrophages possess enhanced antigen presentation properties and lysosome activity, favoring Th1 responses (42). Although there is evidence to the contrary (43), induction of the M1 macrophage activation phenotype is considered beneficial against cancer. M1 exert an anti-tumor effect via direct cancer cytotoxicity through production of reactive oxygen and nitrogen species and/or indirectly via the production of pro-inflammatory cytokines, e.g. interleukin (IL)-12, IL-23 and tumor necrosis factor-α (TNFα), and chemokines [e.g. CCL5, C-X-C Motif Chemokine Ligand (CXCL)5, CXCL9, and CXCL10] and activation of other effector cells (10). Of note, the macrophage-mediated NO-cytotoxity of cancer cells is enhanced by close proximity and cell contact of macrophages to cancer cells via induction of apoptotic mechanisms to the latter, but is independent of the cancer-antigen recognition (44), suggesting the immunotherapeutic potentials of macrophages in “*cold*” tumors. However, the role of classical activated macrophages in tumor cell-specific responses via cytotoxic T cells is equally important, as the levels of the CD8+ T cell-expressing granzyme B were significantly higher in the presence of M1- than M2- polarized TAMs in patients with OSCC (32).

The alternative polarization, induced by Th2-derived cytokines (e.g., IL-4, IL-10, IL-13) that hamper the production of pro-inflammatory cytokines such as TNF-α and IFN-γ, gives rise to the pro-tumor M2 macrophage phenotype (10, 25, 45, 46). M2 macrophages produce anti-inflammatory cytokines, such as IL-1 receptor antagonist, IL-10 and TGF-β, promote the immunosuppressive functions of Foxp3+ regulatory T cells, and enhance the expression of proangiogenic factors (e.g., VEGF) and proteolytic enzymes (e.g., MMPs). Those effects result in the inhibition of anti-tumor immunity mediated by Th1 cells and cytotoxic T cells and, finally, to the escape from immune surveillance (10, 41, 47). The immune escape mechanisms of OSCC might be promoted by the infiltrating TAMs, as a positive correlation has been observed between the levels of CD68+ and CD163+ TAMs and the OSCC cells that expressed the checkpoint PD-L1 protein (48). The simultaneous secretion of IL-10 and expression of the PD-L1 by TAMs has been suggested as a potential mechanism for CD3+ T cell negative regulation by TAMs, identified by the expression of the M2-related markers CD163 and CD204 in OSCC patients (49). M2-polarized TAMs in close proximity of OSCC cells can induce the migration and invasion of the latter, via activation of NF-kB and favor the production of growth factors (e.g., EGF and TGF-β) that promote tumor progression (50). In addition, the expression of the proteins Sonic Hedgehog, Indian Hedgehog and glioma-associated oncogene homolog 1 (GLI-1) in tumor cells and endothelial cells in the OSCC TME, along with the co-expression of Indian Hedgehog ligand in CD163+ TAMS, may be indicative of the role of Hedgehog pathway in promoting neovascularization in OSCC (51).

TAMs are characterized by striking plasticity and except for the M1 and M2 end-stage polarization they may acquire various intermediate activation states, by simultaneously expressing markers related to both M1 (e.g., elevated levels of TNF-α, MMP9, CCL2, CCL5, CXCL9, CXCL10, and CXCL16), and M2 (e.g., increased levels of IL-10, arginase-1, and peroxisome proliferator-activated receptor γ) phenotype (10). As within most solid tumors, the macrophage balance in OSCC tends toward the M2 phenotype (41, 47, 52–54). Co-culture of a monocyte/macrophage like cell line (RAW264.7) with conditioned medium derived from OSCC cell lines stimulated the expression of pro-tumor cytokines and chemokines associated with the M2 phenotype, e.g., IL-10, CCL22, and VEGF-A (54). The receptor for activated C kinase 1 (RACK1) has also been shown to induce OSCC progression, by favoring the polarization toward the M2 phenotype and reducing the levels of M1-phenotype related molecules, such as IL-6, CCL5, and CSF, in an NF-kB axis-dependent manner (55). TAMs-induced VEGF expression in OSCC has also been associated with activation of the TGF-β1/TβRII/Smad3 signaling pathway (56). Okubo et al. (57) developed a xenograft mouse model using a human tongue squamous cell carcinoma (SCC) cell line and reported that the irradiation-induced tumor infiltration by CD11b+ bone marrow-derived cells that acquired an M2-like phenotype, promoted tumor re-vascularization, progression and recurrence after radiotherapy. The potential mechanisms of M2-polarization of TAMs in OSCC also include the OSCC-derived exosomes enclosing micro-RNAs (miR-29a-3p) regulating the activity of SOCS1/STAT6 signals (58), as well as activation of the Axl/PI3K/Akt/NF-kB signaling pathway (52).

The predominance of M1 or M2 phenotype in different subpopulations of OSCC patients has been significantly correlated with the disease outcome (31).

## Prognostic Significance of Macrophages in Disease Outcome

The prognostic significance of TAMs in OSCC has predominantly been evaluated by immunohistochemistry, where the presence of TAMs was correlated with survival parameters, either directly with overall survival and progression/disease/recurrence free survival, or indirectly with clinicopathological factors associated with disease outcome (31, 59). Several TAMs markers have been used, such as the pan-macrophage marker CD68 (both M1 and M2) (30, 35, 47, 54, 56, 60–66), CD204, expressed by dendritic cells and macrophages (54), and markers highly expressed -but not restricted to- M1 (CD11c) (55) or M2 [CD163 (7, 35, 41, 42, 54, 60, 61, 67), CD206 (34, 55)] phenotype.

A growing body of evidence supports the adverse prognostic role of CD68+ TAMs for OSCC patients overall survival (30, 35, 56, 62, 64) or disease-free survival (30, 64, 66). Moreover, elevated numbers of CD68+ TAMs have significantly been associated with high grade (54, 56), increased size (63, 64) and advanced stage (30, 56, 60, 63, 64), lymph node (30, 35, 54, 61, 63, 64) or distant metastasis (47), or high recurrence rate of OSCC (64). In contrast, Wei et al. (65) found that lower mean CD68+ TAMs density was significantly associated with lower differentiation grade and higher clinical stages of OSCC, as well as shorter 5-year survival rate. The latter contrasting findings might be attributed to CD68 staining of both M1 and M2 TAMs, and emphasized the need to identify more specific markers for M1 and M2 subpopulations (65).

Increased levels of CD163+ TAMs, currently considered as an M2-preferable marker, have been correlated with worst overall survival (7, 35, 42, 60, 61, 67) or disease-free survival (7, 67), as well as more frequent recurrence (35) of OSCC. A higher number of CD163+ TAMs has, also, been associated with OSCC poor differentiation (41) and positive lymph nodes (7, 42, 54, 61). A mechanistic explanation for the latter finding was suggested by Yagamata et al. (54), who reported a significant correlation between the number of CD163+ M2 TAMs and the lymphatic vessel density in the TME of OSCC tissue samples, while immunofluorescence analysis revealed the co-expression of VEGF-C in CD163+ TAMs. These results indicated that M2 polarized TAMs may induce lymphangiogenesis through VEGF-C, eventually promoting lymph node metastasis in OSCC (54). Low expression of CD163+ TAMs has also been reported as a negative prognostic factor in OSCC cases presenting high IL-10 and low IFN-γ expression (42). High levels of CD206+ (M2-associated) TAMs have also been correlated with advanced stage, increased tumor size, lymph node metastasis, as well as short disease-free survival (34). Finally, the high M2/M1 ratio, represented by CD206+/CD11c TAMs, showed a significant association with poor disease outcome (55).

The prognostic effects of TAMs in OSCC have, also, been assessed indirectly, through the expression levels of molecules usually secreted by or associated with macrophages. A higher number of CCL2+ cells in the TME, which were found co-expressed in CD163+ TAMs, has been associated with lower 5-year overall survival rates (68). The levels of macrophage migration inhibitor factor (MIF) in OSCC tissues have been adversely correlated with overall and recurrence-free survival, while increased MIF serum concentration is considered indicative for early recurrence (69, 70).

Other immunohistochemical studies have reported a significantly higher expression of CD68+ TAMs (62, 64) or CD163+ M2-polarized macrophages (42, 71) in OSCC compared to oral potentially malignant lesions, e.g., epithelial dysplasia/hyperplasia. TAM markers' expression showed a progressive increase from hyperplasia to low and high degree of dysplasia (64, 72), indicating a possible role of macrophages in promoting the evolution of oral premalignant lesions to invasive OSCC. Finally, significantly increased levels of CD68+ TAMs have been reported in OSCC compared to verrucous carcinoma (73), which is considered less aggressive (74).

In conclusion, an increased immunohistochemical expression of TAMs, predominantly those of M2 polarization, has been associated with adverse OSCC prognosis.

## Targeting Macrophages in Oral Cancer Immunotherapeutics; Lessons Learned From Other Solid Tumor Models

**Figure 1:**
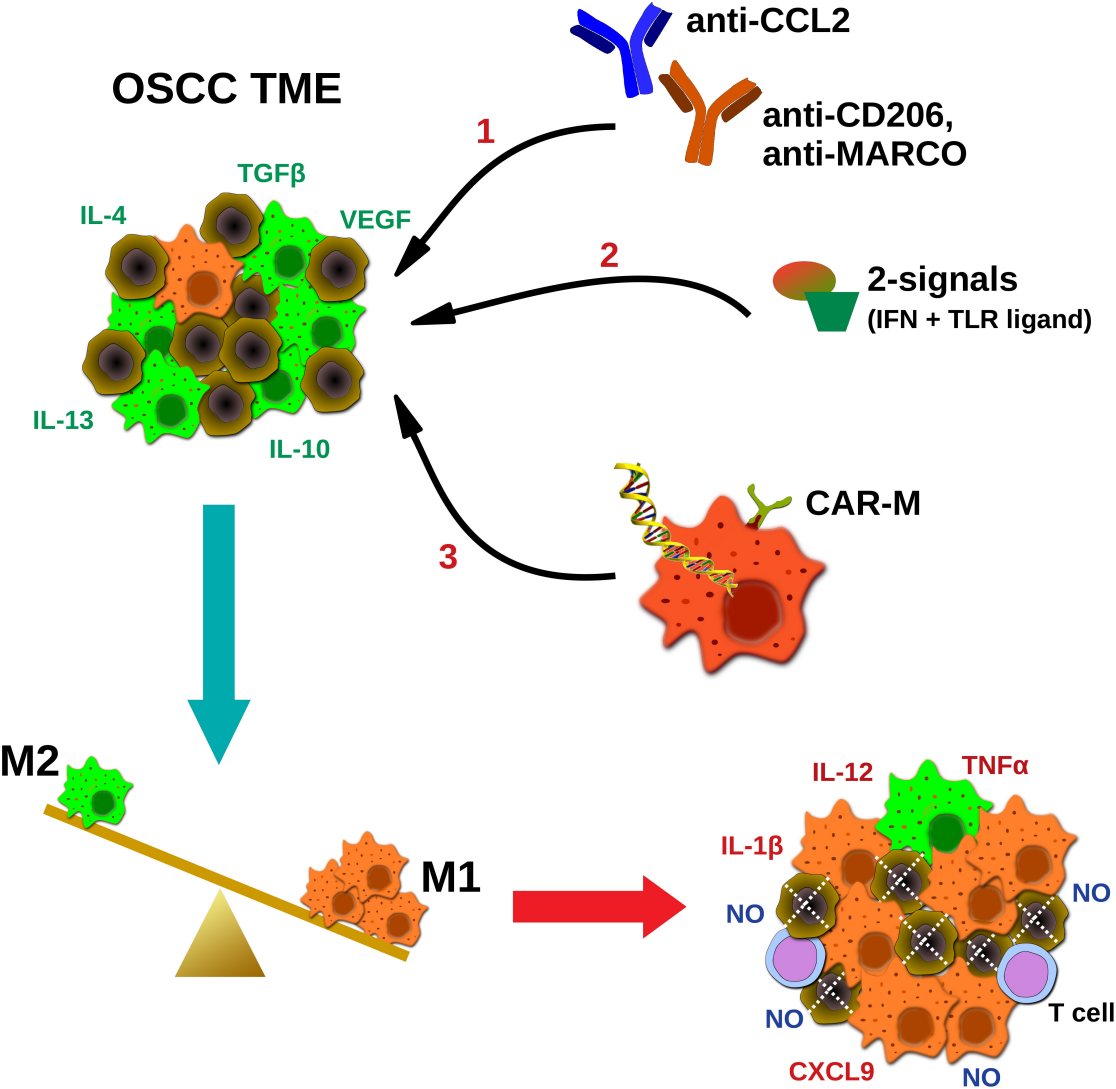
Potential strategies targeting macrophages in oral cancer immunotherapeutics. TAMs in squamous carcinoma microenvironment are abundant and represent an immunosuppressive M2-like phenotype, in favor of tumor progression. Current TAMs-targeting approaches include; (1) blocking antibodies hampering macrophages' infiltration (CCL2) or coating TAMs for destruction using M2-associated markers (CD206, MACRO), (2) delivery of activating stimuli for M2 to M1 repolarization (two signals- IFNs plus TLR ligands), and (3) *ex vivo* generation and infusion of CAR macrophages specifically activated upon cancer antigen recognition. Ultimately, the goal of macrophage-based immunotherapeutics is to reverse the M2 to M1 balance, leading to direct NO-mediated tumor cytotoxicity, and altering of the TME, via secretion of pro-inflammatory molecules by tumoricidal macrophages (M1), and potential activation of other effector cells (e.g., T cells). MARCO, macrophage receptor with collagenous structure.

Given the bipolar role of macrophages in tumor development and their tendency to acquire an immunosuppressive M2-like phenotype during disease progression, current macrophage-based immunotherapeutic approaches aim to alter the M1 to M2 balance in the TME, in favor of the M1. For this purpose several strategies have been developed, including; hampering the monocyte infiltration and macrophage differentiation in the tumor sites (e.g., CCL2 or CSF-1 blocking antibodies), deletion of M2 macrophages in the TME (e.g., antibodies against CD206, scavenger receptors or other M2-associated molecules), or repolarization toward the M1 phenotype and enhancement of their anti-tumor properties (e.g., delivery of activating stimuli and/or antibodies inducing macrophage' phagocytosis, or *ex vivo* macrophage manipulation) (75–77) (Figure 1).

Even though numerous studies point to the strong potentials of macrophage-based immunotherapeutics in various solid tumors, via regulation of the tumor immunity in different levels [reviewed in DeNardo and Ruffell (39)], the direct evidence in OSCC models is yet very limited. In a study using THP-1 human cell line as monocyte-derived macrophage model, pre-activated toward the M1 (IFN-γ+ lipopolysaccharide) or the M2 (IL-4+IL-13) phenotype, it was found that M2 induced the migration and invasion of human tongue SCC cell line HSC-3, when co-cultured with the latter (50). In contrast, using various *in vitro* migration assays, co-cultures with M1 macrophages reduced the invasion of HSC-3 cells, alluding to the anti-tumor properties of this phenotype (50).

Our previous work using primary mouse bone marrow derived macrophages showed that macrophages pre-activated with two simultaneous signals [IFNs plus toll-like receptors (TLRs) ligands] completely blocked the proliferation of Lewis lung carcinoma cell line (LLC), when co-cultured with the latter. Similar results were shown using a macrophage-like cell line (J774.A1) and the mineral-oil induced plasmacytoma cell line (MOPC315), showing the immunotherapeutic potentials of macrophages in different tumor models (78, 79). This macrophage-mediated growth inhibition was shown to be dependent on NO production, since blocking of the latter rescued cancer cell proliferation. Of note, these anti-tumor macrophages secreted significant amounts of pro-inflammatory factors including IL-12p70, TNF-α and the T cell chemoattractant CXCL9 (78, 79), further enhancing the notion for altering the TME and promoting the activation of other effector cells. In this context, recent elegant studies stratifying cutting edge technologies, point to the tumoricidal potentials of macrophages. The CD47-Signal regulatory protein α (SIRPα) axis has gained great interest in immunotherapeutic approaches lately. The interaction of CD47 (also known as “don't eat me” signal) expressed by all cells in the body, with its receptor SIRPα found mainly on macrophages and other phagocytes, hampers the engulfment of the ligand expressing cells (CD47+) by the phagocytes. The upregulation of CD47 is now considered an evoke mechanism (innate immune checkpoint) adopted by cancer cells (80, 81). Alvey et al. (82) showed that *ex vivo* manipulated, SIRPα-inhibited macrophages when systemically injected to mice were able to accumulate into the tumor sites (human lung cancer model), leading to tumor cells engorgement and cancer regression. However, this anti-tumor effect lasted for 1-2 weeks, followed by differentiation of the donor macrophages toward non-phagocytic, high SIRPα TAMs, alluding to the high plasticity of macrophages in response to microenvironmental factors and the need for more permanent approaches. A very recent study, stratifying the chimeric antigen receptor (CAR) technology from T cells, showed that human monocyte-derived CAR engineered macrophages (CAR-M), demonstrated antigen-specific phagocytosis *in vitro*, while a single systemic injection of CAR-M in mice models, significantly reduced tumor burden and improved the overall survival (83). Furthermore, CAR-M demonstrated tumor specific localization in various tumors models and longevity in mice. Intriguingly, they also showed outstanding indirect anti-tumor properties including; secretion of pro-inflammatory molecules, resistance to immunosuppressive cytokines (M1 phenotype persistence), conversion of M2 macrophages to M1, as well as recruitment of- and presentation of antigens to- T cells (83).

Thus, as shown with various other solid tumor models, macrophage-based approaches may hold strong therapeutic potentials. Whether this is also true for OSCC remains to be elucidated.

## Future Perspectives and Conclusions

Advanced OSCC remains practically incurable, as this is reflected on the poor 5-year survival and increased death rates. Although surgical treatment might lead to complete disease management in most of the cases of stage I and II OSCC, cancer recurrence is not rare, while adjuvant chemo-radiotherapy is usually required in advanced stages (4, 5). Given the recent advances in cancer research, the need for implementation of novel diagnostic and treatment tools in its management, has emerged.

TAMs are abundant in the OSCC TME, displaying a bipolar role in disease progression depending on their activation status. A growing body of literature suggests that macrophages may serve as a valid prognostic and therapeutic tool in the arsenal of OSCC treatment modalities. Even though macrophage-based immunotherapeutic research in OSCC is yet inaugural, evidence from various other solid tumor models points to the strong therapeutic potentials of macrophages via inducing direct tumor cytotoxicity and indirectly via altering the immunosuppressive TME and induction of a systemic anti-tumor immunity and activation of other effector cells. Identification of novel M1- and M2- specific markers and further experimentation implying macrophages' tumoricidal activation in OSCC models, might close the gap from bed-to-bedside, leading to the clinical implementation of novel macrophage-based strategies, in prognosis and immuno-treatment of this cancer type.

## Author Contributions

EMK performed the literature research and wrote the manuscript. KT contributed to manuscript writing and formatting. PC conceived the idea, designed the lay out, wrote and reviewed the manuscript. All authors have approved the final draft.

## Conflict of Interest

The authors declare that the research was conducted in the absence of any commercial or financial relationships that could be construed as a potential conflict of interest.

## Abbreviations

Akt: Protein kinase B
CAR: chimeric antigen receptor
CAR-M: CAR engineered macrophages
CCL: chemokine (C-C motif) ligand
CD: cluster of differentiation
CSF-1: colony stimulating factor-1
CXCL: C-X-C Motif Chemokine Ligand
EGF: epidermal growth factor
GLI-1: glioma-associated oncogene homolog 1
ICB: immune checkpoint blockade
IFN-γ: interferon-γ
IL: interleukin
iNOS: inducible nitric oxide synthase
MHCI: Major Histocompatibility Complex (MHC) Class I
MIF: migration inhibitor factor
MMP: matrix metalloproteinase
NF-kB: nuclear factor kappa beta
NO: nitric oxide
OSCC: oral squamous cell carcinoma
PD-L1: programmed death-1 ligand 1
PI3K: phosphatidylinositol 3-kinase
RACK1: receptor for activated C kinase 1
SIRPα: Signal regulatory protein α
SOCS1: suppressor of cytokine signaling 1
STAT6: signal transduction and transcriptional activator 6
TAMs: tumor-associated macrophages
TGF-β: transforming growth factor beta
TβRII: type II TGF-β receptor
Th: T helper type
TILs: tumor infiltrated lymphocytes
TLR: toll-like receptor
TME: tumor microenvironment
TNF-α: tumor necrosis factor-α
VEGF: vascular endothelial growth factor.
